# A diffusion-conditioned representation learning framework for disease classification in medical imaging

**DOI:** 10.1186/s13104-026-07858-8

**Published:** 2026-07-19

**Authors:** Jutika Borah, Rajkumar Saini, Abdur R. Fayjie, Debarun Chakraborty, Hidam Kumarjit Singh

**Affiliations:** 1 Gauhati University, Guwahati, 781014 India; 2 Luleå University of Technology, 97187 Luleå, Sweden; 3 KU Leuven, 3000 Leuven, Belgium; 4 Tezpur University, Tezpur, 784028 India

**Keywords:** Medical image classification, Representation learning, Model calibration, Diffusion model, Uncertainty quantification, Robustness

## Abstract

**Objective:**

Recently, deep learning models in medical imaging have undergone tremendous advancements. However, these deterministic discriminative models often tend to overfit and produce overconfident predictions. To explore a more efficient strategy, we propose a diffusion-conditioned representation learning that leverages the internal dynamics of a diffusion model to extract noise-level awareness and diverse semantic representations in disease classification and detecting distributional shifts. The proposed method inherently encodes aleatoric uncertainty by sampling features from multiple denoising time steps, which is then used to regularize prediction during training via entropy regularized loss.

**Result:**

We conducted extensive experimentation over five diverse clinical datasets, and our framework demonstrates competitive performance with reduced prediction entropy and improved robustness to input degradation. The negative entropy–accuracy correlation, expected calibration error, and dynamic uncertainty demonstrate the effectiveness of the proposed framework in classification tasks. Our framework achieved competitive performance compared with traditional convolutional neural networks and vision transformers with an AUC of 83.81%, 97.45%, 86.15% and 96.75% on datasets 1, 2, 3 and 4, respectively. The reduced ECE values on the datasets demonstrate the calibrated performance of the proposed method.

## Introduction

In modern healthcare, with the increasing use of digital medical imaging, medical image analysis has become a crucial part of accurate clinical treatment planning [1]. Digital medical imaging has automated image classification essential for efficient clinical decision-making. Although CNNs and ViTs have shown promising results in medical imaging, they require large labeled datasets and struggle with domain specific challenges of low-contrast lesions, blurry effects, and texture variations, highlighting the need for modeling rich in representation of the fetal region [2–8].

Diffusion models including latent diffusion models (LDMs) and denoising diffusion probabilistic models (DDPMs) have demonstrated success over GANs and VAEs by learning to reverse a Gaussian noise process in a compressed latent space, allowing superior image quality, diversity, and hierarchical visual understanding [9–12]. Their ability to capture complex data distribution has made them highly effective for representation learning and downstream classification tasks, including medical imaging [13].

While diffusion models are capable of generating rich hierarchical representations through pretrained U-Net activations, their generalization under domain shifts and multi-scale pathological variation remains limited compared to supervised backbones such as ViTs or ResNets. The pioneering DiffMIC [14] and DiffMIC-v2 [15] use diffusing label priors alongside image features and iteratively denoising predictions via a fine-tuned U-Net for classification. Despite recent advances, most frameworks treat diffusion models and classifiers as separate entities, leaving the direct exploitation of frozen diffusion representation for uncertainty-aware, discriminative task largely underexplored.

We propose a diffusion-conditioned framework that repurpose diffusion models as representation priors via stochastic multi-timestep feature extraction, rather than generative priors. Operating entirely in latent space with a frozen backbone, our approach addresses discriminative representation learning while incorporating intrinsic uncertainty quantification for improved medical image classification.

## Materials and methodology

### Datasets

In this study, we evaluated the proposed framework using five publicly available medical imaging datasets. We randomly split the data into two main sets, where 60% of the data were used for training, and the remaining 40% were used for evaluation as a test set. A validation subset is further separated from the test data (20% validation and 20% test) and used exclusively for hyperparameter tuning. Each dataset was used to train and evaluate the model independently to ensure a fair comparison and avoid any data leakage across datasets.

(i) *Dataset-1:* The database [16] consists of chest radiographs for COVID-19-positive patients, including images of normal and viral pneumonia patients. There were 3,616 COVID-19 positive patients, 6,012 patients with lung opacity, 10,192 normal controls, 1,345 patients with viral pneumonia, and corresponding lung masks.
(ii) *Dataset-2:* The images in the Shenzhen dataset [17] were composed in collaboration with Shenzhen No.3 People’s Hospital, Guangdong Medical College, Shenzhen, China. The dataset contains 662 frontal chest X-rays, of which 326 are normal cases, and 336 are cases with TB cases.
(iii) *Dataset-3:* The pneumonia chest X-ray dataset [18] comprises 5,856 CXR images categorized into two classes: pneumonia and normal. This dataset was collected from retrospective cohorts of pediatric patients at the Guangzhou Women and Children’s Medical Center in Guangzhou, China.
(iv) *Dataset-4:* The LC25000 dataset [19] is a histopathology image dataset with 25,000 color images of 768 × 768 resolution in .jpeg file format with 5 classes comprising lung adenocarcinoma, benign lung tissue, lung SCC, colon adenocarcinoma, and benign colonic tissue.
(v) *Dataset-5:* The CheXpert [20] is a large chest X-ray dataset retrospectively collected from Stanford Hospital, USA with approximately 224,316 chest X-rays of 65,240 patients with both frontal and lateral views. These classes are completely disjoint from all other datasets used in the study, and so taken as an out-of-distribution dataset.

**Fig. 1:**
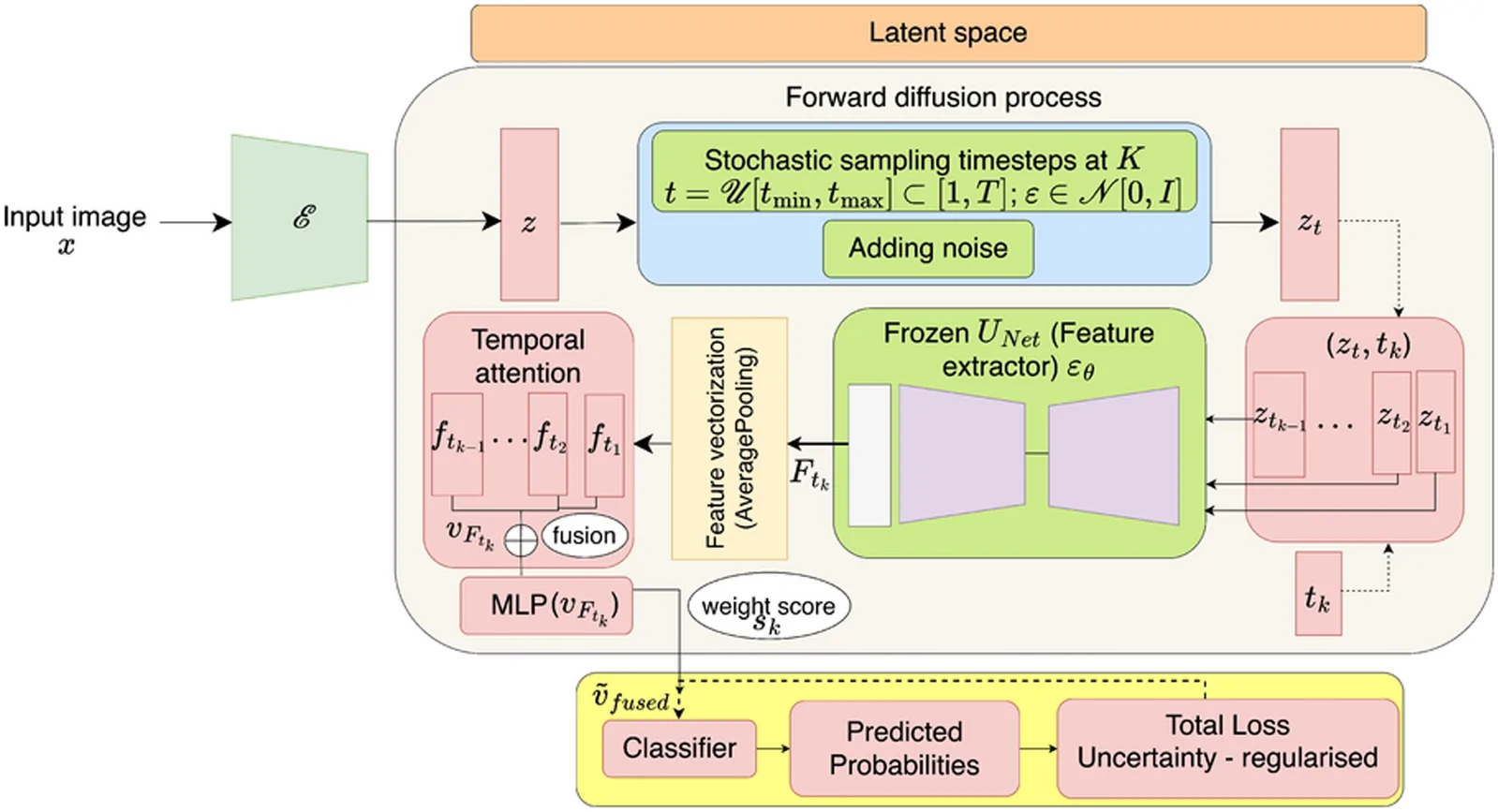
An overview diagram of the proposed diffusion-conditioned framework, where the VAE transforms image data from the pixel space into the latent space, and a forward diffusion process adds noise in this latent space

### Proposed methodology

Given an input image $\boldsymbol{x}$, we first project it into a lower-dimensional latent space via an encoder which, in our proposed framework as illustrated in Fig. 1, is a pretrained VAE encoder $\mathcal {E}$. In the forward stage, $\boldsymbol{x}$ is gradually transformed into a noisy representation through a Markov chain. The latent representation $\boldsymbol{z}$ is obtained as:

1 $$\begin{aligned} \boldsymbol{z} = \text {scale} \cdot \mathcal {E}(\boldsymbol{x}), \end{aligned}$$

where the scale factor is set to maintain consistency with the diffusion model’s training distribution.

#### Stochastic multi-timestep feature extraction

Simulating a forward diffusion process with *K* stochastic forward passes produces intermediate feature embeddings at multiple denoising layers. This enables the encoder to capture multiscale semantic information. Unlike standard DDPM [12] inference, which iteratively denoises $x_{T}$ to recover the initial input image $x_0$, our framework performs *K* parallel stochastic passes. For each pass $k \in \{1, \dots , K\}$, we sample a random timestep $t_k$ from a predefined range $[t_{min}, t_{max}]$. Gaussian noise was introduced into the latent representation $\epsilon \sim \mathcal {N}(0, I)$ according to the forward diffusion schedule:

2 $$\begin{aligned} \boldsymbol{z}_{t_{k}} = \sqrt{\bar{\alpha _t}}\boldsymbol{z}_0 + \sqrt{1 - \bar{\alpha _t}}\epsilon , \end{aligned}$$

where $\bar{\alpha _t}$ represents the cumulative product of the noise variance schedule.

These noisy latents $z_{t_k}$ are processed by a frozen U-Net $U_{net}$. High values of $t_k$ force the encoder to extract the global anatomical context, whereas low values of $t_k$ allow the extraction of fine-grained pathological textures. The features were extracted from *K* intermediate time steps $\{t_k\}^K_{k=1} \subset \mathcal {U}[t_{min}, t_{max}]$:

3 $$\begin{aligned} F_{t_k} = \textrm{Flatten}\!\left( U_\text {net\_Encoder}(\boldsymbol{z}_{t_k}) \right) \in \mathbb {R}^{d}. \end{aligned}$$

These features capture progressive refinement, i.e., early timesteps $(t \sim t_{max})$ reflect coarse noise-robust structures, and late timesteps $(t \sim t_{min})$ encode fine details. Each feature map $F_{t_{k}}$ is then collapsed into feature vector $v_{F_{t_{k}}} \in \mathbb {R}^D$ via the global average pooling and a trainable projection layer $g_\theta$:

4 $$\begin{aligned} \boldsymbol{v}_{F_{t_{k}}} =g_{\theta }\!\left( \operatorname {AvgPool}\!\left( F_{t_k}\right) \right) . \end{aligned}$$

To effectively aggregate features from *K* different noise levels, we integrate a temporal attention mechanism. This mechanism ensures that the model focuses on the timesteps most relevant to the specific pathology being analyzed, acting as a dynamic filter. A multi-layer perceptron (MLP) computes an importance score $s_k$ for each feature vector:

5 $$\begin{aligned} \boldsymbol{v}_{F_{t_{k}}}: s_{k} = \text {MLP}\!\left( \boldsymbol{v}_{F_{t_{k}}}\right) . \end{aligned}$$

The final fused representation $\tilde{v}_\text {fused}$ is calculated as a weighted sum:

6 $$\begin{aligned} w_k = \frac{\operatorname {exp}(s_k)}{\sum ^K_{j=1}\operatorname {exp}(s_j)}, \tilde{\boldsymbol{v}}_\text {fused} = \sum ^K_{k=1}w_k \boldsymbol{v}_{F_{t_{k}}}. \end{aligned}$$

Instead of just averaging the features from all *K* passes, it assigns a weight or importance score to each pass.

#### Classifier head with uncertainty-regularized training

Instead of noise estimation, we utilize a combination of negative log-likelihood (NLL) and entropy regularization. The fused feature $\tilde{v}_\text {fused}$ is passed through a classifier head:

7 $$\begin{aligned} h_{\psi }: p(y \mid \boldsymbol{x}) = \textrm{Softmax}\!\left( h_{\psi }(\tilde{v}_\text {fused})\right) . \end{aligned}$$

To prevent overconfidence, we minimize the uncertainty-regularized loss:

8 $$\begin{aligned} \mathcal {L}_\text {reg} = \text {NLL}(y, p{(y \mid \boldsymbol{x}})) + \lambda \cdot \mathcal {H}(p(y \mid \boldsymbol{x})), \end{aligned}$$

where,

9 $$\begin{aligned} \mathcal {H}(p(y \mid \boldsymbol{x})) = -\sum ^C_{c=1}p_c\log p_c, \end{aligned}$$

which is Shannon and represents predictive entropy, and hyperparameter *λ > 0* which is empirically set to 0.1 controls the calibration strength. *λ * was selected based on preliminary validation experiments to balance classification accuracy and calibration performance.

At the test time, we sample *M=10* timesteps $\{\, t_{k}^{(m)} \,\}_{m=1}^{M}$ uniformly from $[t_{min}, t_{max}]$, Computing $p^{(m)} (y \mid \boldsymbol{x})$ for each, return mean prediction:

10 $$\begin{aligned} \bar{p}(y \mid \boldsymbol{x}) = \frac{1}{M} \sum _mp^{(m)}. \end{aligned}$$

reporting predictive entropy $\mathcal {H}(\bar{p})$ as an uncertainty metric.

During the training process, in each iteration a timestep *t* is sampled from a uniform distribution $t \sim \mathcal {U}[t_{min}, t_{max}] \subset [1, T]$. We employ a linear variance schedule with $\beta _{1} = 1 \times 10^{-4}$ and $\beta _{T} = 0.02$ to inject Gaussian noise where it is interpolated from $\beta _t$ to $\beta _T$. The noise intensity is determined by this variance of perturbation applied across *K* discrete stochastic timesteps simulating the varying levels of degradation in data. We used a frozen backbone (SD v1.5) as the encoder. The features are extracted from the final output of the last down-sampling block, which has the high-level feature map before the bottleneck of the frozen $U_{net}$. The feature map is then averaged globally to obtain a vector of fixed dimension, and is projected subsequently to the target feature dimension.

During the inference, our proposed framework employs a parallel stochastic scheme, where for a single input image $\boldsymbol{x}$, we perform *K* parallel forward passes. In each pass, a different random noise $\epsilon _{k}$ and timestep $t_{k}$ are applied. The final classification $\hat{y}$ is derived from the weighted average of the probability distributions across all *K* passes. The predictive entropy across these *K* passes is calculated.

## Experiments

All the implementations and experiments are performed via the PyTorch library. The batch size for training is set to 8. The maximum number of epochs is set to 50, and the learning rate is initialized to $3 \times 10^{-4}$ with a weight decay of *1e-4*. We chose Adam as the optimizer for the training stage. For a fair comparison, all the baseline models were trained under identical training settings, including optimizer configuration, learning rate and data splits. All experiments were conducted using a fixed random seed to ensure reproducibility of the reported results.

### Evaluation metrics

For a comprehensive evaluation, we employed standard performance metrics such as accuracy, area under the receiver operating characteristic (ROC) curve, area under the Curve (AUC), the area under the precision-recall (AUPR), precision, sensitivity, specificity, the F1 score, the true positive rate (TPR), and the false positive rate (FPR). For uncertainty quantification, we used entropy [21] and expected calibration error (ECE) [22, 23] as the evaluation metrics. ECE measures the correspondence between probabilities predicted and accuracy [24]. As mentioned by [25], these metrics are reliable for comparing out-of-distribution detection. To determine statistical significance, we used the Pearson correlation value *r* and the significance value *ρ *.

**Table 1:** Model performance metrics across four datasets

Dataset	Accuracy	AUC	AUPR	Precision	Sensitivity	Specificity
Dataset 1	0.7559	0.8381	0.7284	0.6633	0.6704	0.8288
Dataset 2	0.9500	0.9745	0.9634	0.9300	0.8986	0.8980
Dataset 3	0.8436	0.8615	0.8706	0.7800	0.6768	0.6213
Dataset 4	0.9271	0.9675	0.9256	0.9400	0.8900	0.8456

**Table 2:** Additional calibration and uncertainty metrics across four datasets

Dataset	Accuracy	F1-score	ECE ↓	Correlation
Dataset 1	0.7559	0.6666	0.0677	− 0.3892
Dataset 2	0.9500	0.9129	0.0224	− 0.4536
Dataset 3	0.8436	0.7247	0.2161	− 0.4433
Dataset 4	0.9271	0.9225	0.1226	− 0.5034

The more negative the results, the better the Pearson correlation between entropy and accuracy

## Results and analysis

**Fig. 2:**
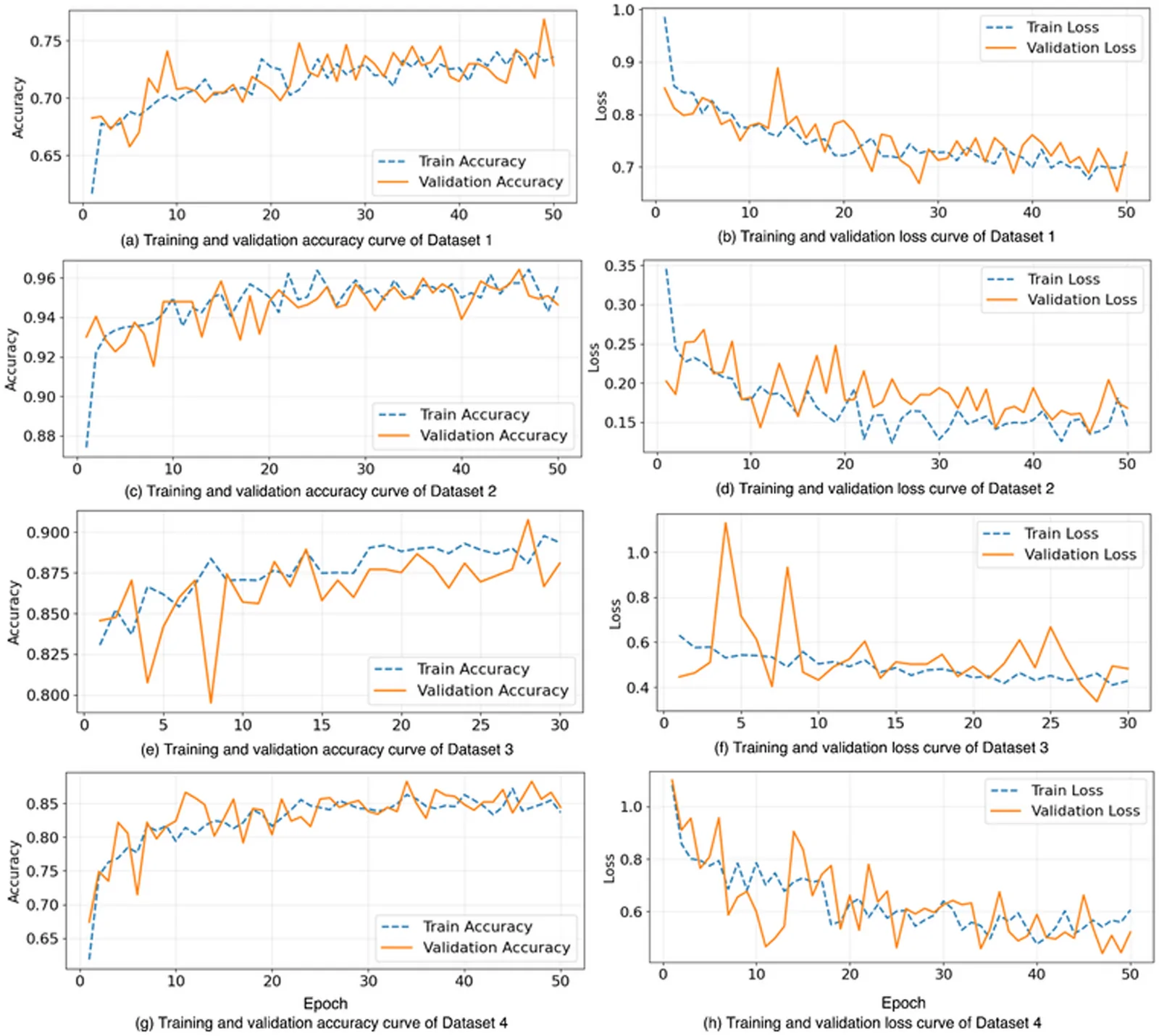
Training and validation learning curves across four datasets

**Fig. 3:**
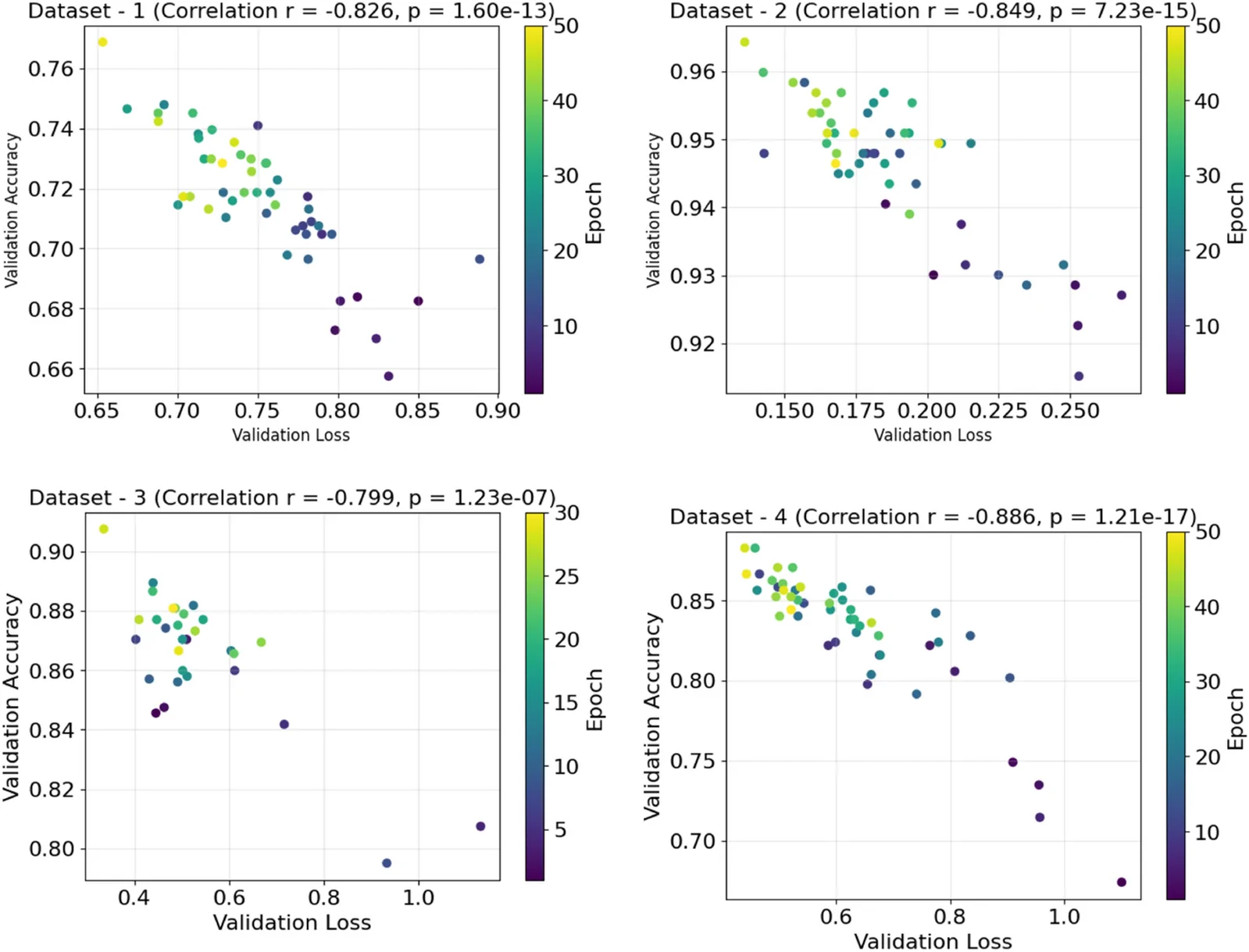
Scatter plots demonstrating the correlation between validation accuracy and loss across the datasets

**Fig. 4:**
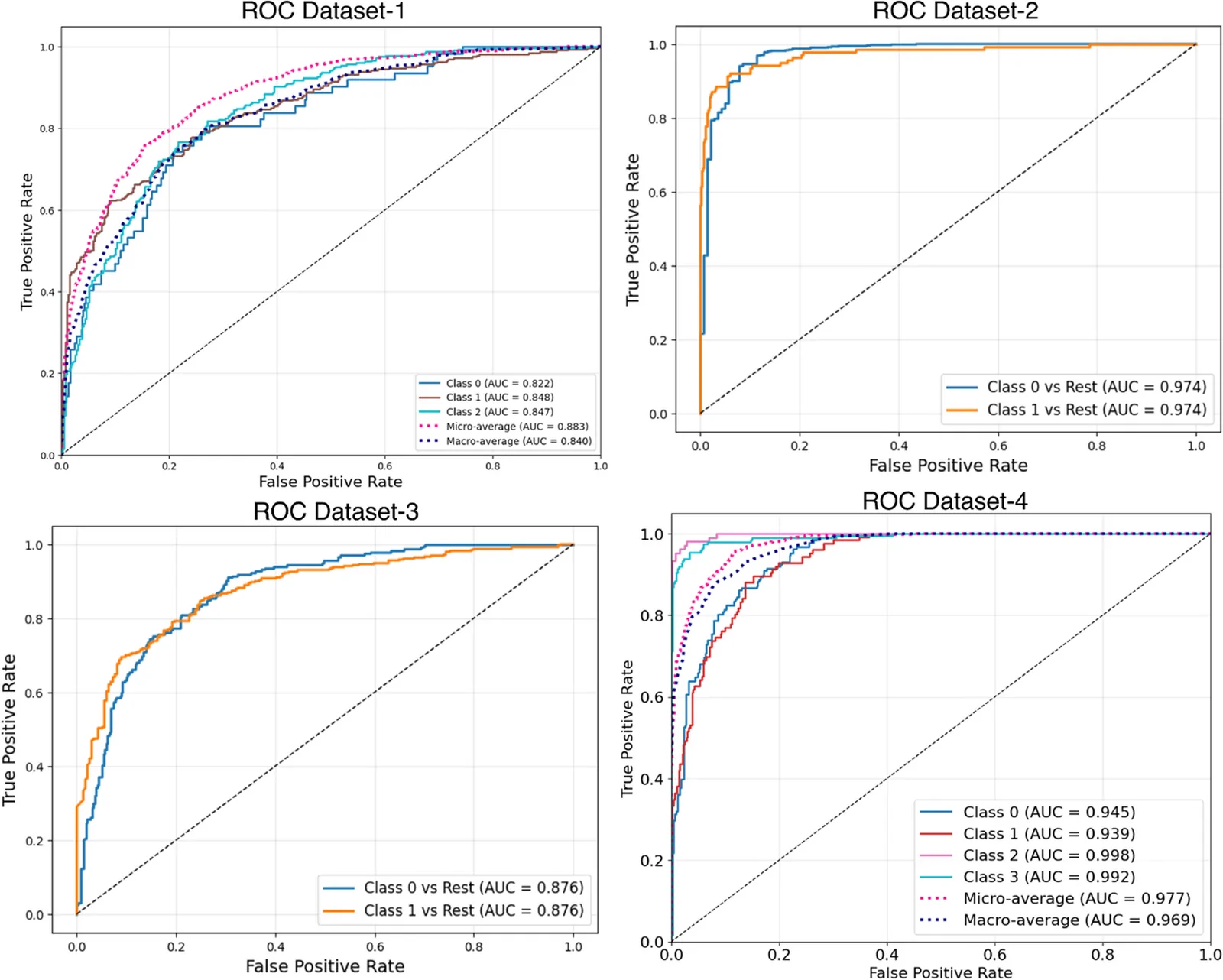
ROC curves for four datasets

The results with performance evaluation metrics are reported in Table 1. The corresponding ROC curves are shown in Fig. 4. The behavior of training dynamics in all datasets observed in Fig. 2, demonstrates minimal accuracy gaps, indicating reduced overfitting and stable generalization. The observed statistically significant negative correlation results between the validation accuracy and loss in Fig. 3 across four datasets with $$ \begin{gathered} r = - 0.826,p = 1.60 \times 10^{{ - 13}} ; \hfill \\ r = - 0.849,p = 7.23 \times 10^{{ - 15}} \hfill \\ ;r = - 0.799,p = 1.23 \times 10^{{ - 7}} \hfill \\ \end{gathered} $$ respectively, demonstrate the stable optimization dynamics. Table 3 explicitly demonstrates the performance in out-of-distribution data detection.

The negative Pearson correlation between entropy and accuracy reflected in Tables 2 and 3 indicates that higher model uncertainty is associated with increased prediction errors. The entropy values observed indicate that efficient signaling of uncertainty occurs when faced with unfamiliar data. These observations support the reliability of the proposed framework. With a completely disjoint set of distributions, the framework achieves higher ECE values, which shows that our framework flags “what it does not know" instead of producing overconfident predictions desirable for safety-critical medical applications.

### Ablation study

The results of our ablation study, presented in Tables 4 and 5, effectively demonstrate the performance of the proposed framework in comparison to its individual components and state-of-the-art methods. The first baseline for evaluation is a vanilla network, which does not have a diffusion process or uncertainty-regularized training involved in the network. The vanilla network consists of a simple CNN architecture. In the next step, we added the diffusion process by introducing noise into the first step and further added UNet without attention to build another baseline network for image classification. Simple diffusion denotes baseline with a single random sample timestep without any temporal attention or multiple-timestep fusion.

Table 5 compares the proposed framework with other supervised DL methods. For independent quantitative comparison, we took ResNet-50 and ViT-B/16, and evaluated them on the held-out test set using their corresponding trained linear classifier. The same training, validation, and test split was used across all backbones to ensure fair comparison. Overall, the ablation results indicate that both stochastic diffusion features and entropy regularization jointly contribute to improved predictive performance and calibration.

**Table 3:** Performance comparison of predictive uncertainty, confidence, calibration error, and Pearson correlation between entropy and accuracy across training-evaluation datasets

Dataset	Entropy ↑	ECE ↑	Correlation	AUC	AUPR	FPR	TPR
Training on dataset-2, evaluation on dataset-3							
Dataset 2 → 3	0.2713	0.5641	− 0.0428	0.6160	0.6072	0.4868	0.5132
Training on dataset-3, evaluation on dataset-2							
Dataset 3 → 2	0.2450	0.6748	− 0.0404	0.7924	0.7023	0.4671	0.5329
Training on dataset-2, evaluation on dataset-5							
Dataset 2 → 5	0.3863	0.6403	0.1170	0.3461	0.2416	0.2016	0.2164
Training on dataset-3, evaluation on dataset-5							
Dataset 3 → 5	0.3023	0.6486	− 0.0486	0.5057	0.2327	0.3986	0.4127

→ in the dataset column indicates that the model trained on one dataset is evaluated on a different dataset

**Table 4:** Extensive experiments on the effectiveness of the overall framework compared to baselines with Dataset-2

Metrics	Vanilla network	Simple diffusion	UNet without attention	Ours
Accuracy	0.9023	0.9011	0.9267	0.9500
AUC	0.9345	0.9321	0.9567	0.9754
Entropy	0.4567	0.2456	0.2466	0.1325
Correlation	0.5643	− 0.5262	− 0.5098	− 0.4536

**Table 5:** Quantitative comparison of the proposed framework with state-of-the-art methods on classification tasks

Metric	Dataset-1			Dataset-2			Dataset-3		
	ResNet-50	ViT-B/16	Ours	ResNet50	ViT-B/16	Ours	ResNet50	ViT-B/16	Ours
Accuracy	0.7379	0.7596	0.7559	0.9423	0.9537	0.9548	0.7462	0.7692	0.8436
F1-score	0.6689	0.6688	0.6666	0.9045	0.9098	0.9129	0.7174	0.7010	0.7247
Entropy	0.4678	0.3224	0.3587	0.2978	0.2113	0.1139	0.4985	0.4567	0.2228

## Discussion

The proposed work presents a stochastic feature encoding representation learning framework for medical image classification, where it can adaptively prioritize multiscale information. Furthermore, it is demonstrated that the inherent stochasticity introduced in the diffusion process can be effectively utilized for uncertainty estimation. Through a regularized entropy, the framework provides calibrated predictions. The experimental results observed with different datasets indicate that the proposed approach showed competitive performance compared to state-of-the-art supervised methods such as ResNet and ViT. This is a unified framework that integrates stochastic diffusion process and uncertainty regularized training for reliable and interpretable DL in medical image classification. Across multiple corruption levels, the framework learns noise-conditioned representations.

The use of entropy-accuracy correlation provides a level of safety for AI-based diagnostic tools, where low entropy indicating high confidence can be sent for routine clinical review, whereas high entropy cases, which often involve poor image quality, unknown and rare pathologies, etc., can be forwarded for re-evaluation by a radiologist. This will optimize the time and reduce the risk of oversight in ambiguous cases. Thus, from a clinical safety perspective, efficient out-of-distribution detection is a crucial step where overconfident misinterpretation poses a substantial risk in clinical diagnostic decision-making. In Fig. 3, the observed tight clustering of points in later epochs in the low-loss and high-accuracy further validates the controlled training progression with the absence of diverging validation and reduced overfitting.

The lower values of ECE ensure that the probability scores generated by the proposed framework are not overconfident. The calibration error on out-of-distribution data can be used as a consensus that aligns with the reasoning of clinical Bayesian. With ECE, sample-level out-of-distribution detection cannot be generated; however, the increase in calibration error under such shifts highlights the existence of risk of unreliable confidence in unseen clinical settings. Comparing ECE across different approaches, it is observed that while all approaches achieve similar low calibration errors under a mild shift in distribution between the training and testing data, a pronounced difference emerges when the methods are evaluated on disjoint sets. The proposed framework consistently responds to distribution shifts by elevating the predictive entropy, thereby explicitly signaling distributional mismatches in the disease classification task, important in safety-critical environments. Overall, the findings indicate that uncertainty-aware diffusion representations may enhance the reliability of medical image classification systems under distributional variability.

### Limitations

Despite these promising results, our work also has several limitations that should be acknowledged, and it has several avenues for future exploration. While promising, these findings should be interpreted as preliminary evidence toward a clinically reliable decision-support system. The dataset used in the study may not fully capture real-world variables, which should be considered when interpreting generalizability. Second, the reported strong loss-accuracy correlations have confirmed the stability of the training process; however, further evaluations are required on a broad range of distributional shifts, including diverse image acquisition conditions and rare pathologies. Third, although the proposed framework demonstrated meaningful uncertainty estimation, incorporating post-hoc techniques such as conformal prediction would further enhance probabilistic reliability, particularly for applications requiring explicit error control. Expanding the validation to patient-specific or slice-level uncertainty and multi-center cohorts would further strengthen the applicability of the proposed framework. In the future, we aim to address these concerns through further refinement and validations

## Conclusion

By shifting from iterative denoising to a parallel, multi-timestep extraction strategy, we effectively capture rich hierarchical features. The experimental results demonstrate improved model calibration and reliability, showing competitive performance compared to standard supervised baselines. The proposed framework appears to respond to distribution shifts thereby explicitly signaling distributional mismatches in the classification task.

## Data Availability

The datasets used and/or analyzed during the current study are publicly available or available from the original corresponding author upon reasonable request.
